# Endoscope-enhanced fluorescence-guided microsurgery increases survival in patients with glioblastoma

**DOI:** 10.1007/s00701-023-05862-6

**Published:** 2023-11-11

**Authors:** Christoph Bettag, Bawarjan Schatlo, Tammam Abboud, Daniel Behme, Christoph Bock, Christian von der Brelie, Veit Rohde, Dorothee Mielke

**Affiliations:** 1 Department of Neurosurgery, University Hospital Goettingen, Goettingen, Germany; 2 Department of Neuroradiology, University Hospital Goettingen, Goettingen, Germany; 3 Department of Neuroradiology, University Hospital Magdeburg, Magdeburg, Germany; 4 Department of Neurosurgery, Johanniter Hospital Bonn, Bonn, Germany

**Keywords:** 5-Aminolevulinic acid, Endoscope, Fluorescence-guided surgery, Glioblastoma

## Abstract

**Purpose:**

Extent of resection (EOR) predicts progression-free survival (PFS) and may impact overall survival (OS) in patients with glioblastoma. We recently demonstrated that 5-aminolevulinic acid-(5-ALA)-fluorescence-enhanced endoscopic surgery increase the rate of gross total resection. However, it is hitherto unknown whether fluorescence-enhanced endoscopic resection affects survival.

**Methods:**

We conducted a retrospective single-center analysis of a consecutive series of patients who underwent surgery for non-eloquently located glioblastoma between 2011 and 2018. All patients underwent fluorescence-guided microscopic or fluorescence-guided combined microscopic and endoscopic resection. PFS, OS, EOR as well as clinical and demographic parameters, adjuvant treatment modalities, and molecular characteristics were compared between microscopy-only vs. endoscopy-assisted microsurgical resection.

**Results:**

Out of 114 patients, 73 (65%) were male, and 57 (50%) were older than 65 years. Twenty patients (18%) were operated on using additional endoscopic assistance. Both cohorts were equally distributed in terms of age, performance status, lesion location, adjuvant treatment modalities, and molecular status. Gross total resection was achieved in all endoscopy-assisted patients compared to about three-quarters of microscope-only patients (100% vs. 75.9%, *p*=0.003). The PFS in the endoscope-assisted cohort was 19.3 months (CI95% 10.8–27.7) vs. 10.8 months (CI95% 8.2–13.4; *p*=0.012) in the microscope-only cohort. OS in the endoscope-assisted group was 28.9 months (CI95% 20.4–34.1) compared to 16.8 months (CI95% 14.0–20.9), in the microscope-only group (*p*=0.001).

**Conclusion:**

Endoscope-assisted fluorescence-guided resection of glioblastoma appears to substantially enhance gross total resection and OS. The strong effect size observed herein is contrasted by the limitations in study design. Therefore, prospective validation is required before we can generalize our findings.

## Introduction

Despite recent advances, the survival of patients with glioblastoma multiforme (GBM) remains poor. Surgical resection is a cornerstone of GBM treatment, and a greater extent of resection (EOR) has been associated with better progression-free survival (PFS) and overall survival (OS) in several studies [1, 5, 11]. Complete removal of the contrast-enhancing tumor tissue, as seen in magnetic resonance imaging (MRI), while preserving neurological function is the main surgical goal. 5-aminolevulinic acid (5-ALA) is a porphyrin generating fluorescence in GBM which can be visualized under blue light of the microscope [13]. Microscopic fluorescence-guided resection has been proven to increase the rate of gross total resection (GTR) from 36 to 65% [12]. This conversely means that in more than a third of patients, GTR is not achieved despite being intended and feasible. This finding might be related to the limitations of microscopic fluorescence-guided resection: Visible fluorescence of tumor tissue depends on cell density and cellular metabolism [6], as well as on adequate exposure of tumor tissue towards blue light. Visualization of fluorescence might be difficult due to collapsing resection margins and decreasing illumination with increasing depth of the resection cavity. Our group recently demonstrated that the assistance of an endoscope with a white and a blue light source, being capable of inducing protoporphyrin IX (PpIX) fluorescence, allows overcoming some limitations of fluorescence-guided microscopic tumor resection [2–4]. However, it is hitherto unknown whether endoscope-assisted fluorescence-guided GBM resection affects survival. This is the first study comparing the impact of microscopic versus combined microscopic/endoscopic-fluorescence guided surgery on survival in GBM patients.

## Methods

We performed a retrospective single center analysis of patients with primary GBM, which underwent routine microscopic fluorescence-guided resection between January 2011 and February 2018. Between January 2015 and April 2018 an endoscope with a white and blue light source was available and used in addition to the microscope. Patients were recorded for PFS and OS, as well as for demographic parameters (age, sex), performance status (Karnofsky performance scale [KPS]), molecular status (O 6-methylguanine-DNA methyltransferase [MGMT]), numbers of recurrence and surgeries, and adjuvant treatment modalities (Stupp protocol, radiation alone, bevacizumab, lomustine, tumor-treating-fields [TTF], procarbazine/lomustine/vincristine [PCV]).

The study was reviewed by the local ethics committee (approval number 1/12/22). All patients were informed in case of the application of the endoscope and written consent was obtained within the standard informed consent process for surgery from all patients.

### Inclusion and exclusion criteria

Only patients with primary GBM presumed to be non-eloquently located were included to ensure that GTR could be achievable in all patients. Patients undergoing biopsy or requiring intraoperative neuromonitoring (considered as a surrogate parameter for eloquent location) were excluded. Patients without postoperative MRI within 48 h were also excluded.

### Surgical protocol

A standard dose of 5-ALA of 20 mg/kg was administered 4 h prior to surgery. A standard neuronavigated microscopic fluorescence-guided resection (OPMI Pentero® 800, Carl Zeiss, Oberkochen, Germany) was performed. In case of combined endoscopic/microscopic surgery, the resection cavity was scanned using an endoscope (Hopkins II, 4 mm, viewing angle 0 degrees, KARL STORZ, Tuttlingen, Germany) with a special light source (D-light C; KARL STORZ) and a camera system (Tricam SL II; KARL STORZ) after complete microscopic removal of all visualized fluorescent tissue (solid and vague) [2]. The D-light allows switching between the white light and the blue light source mode by means of an appropriate band-pass filter in the light transmission path. A long-pass filter at the eyepiece of the endoscope blocks the excitation light which enables the detection of fluorescence signals from the tumor cells. The excitation and detection filter system allow enough blue light to be transmitted so that the red fluorescence from the endogenous fluorochromes and nonspecific PpIX fluorescence is suppressed, causing the normal tissue to be visualized as blue [10]. Microscopic fluorescent tissue and endoscopic fluorescent tissue, being not visualized by the microscope, were completely removed and embedded separately for histopathological examination.

### Postoperative assessment

All patients underwent MRI within 48 h after surgery. Any residual contrast-enhancement > 0.175 cm^3^ was defined as residual tumor. Performance status was evaluated using the Karnofsky performance scale at the time of discharge. All included patients regularly underwent clinical assessment and contrast-enhanced MRI follow-up every three months. Recurrence was defined on follow-up MRI according to the Response assessment in neuro-oncology criteria (RANO) criteria by an experienced neuroradiologist [14].

### Statistical analysis

The entire analysis was performed using SPSS (version 23, IBM Corp.). Continuous variables were measured as mean or median values and standard deviation. Differences between both cohorts were analyzed using unpaired *t*-test. Descriptive survival was analyzed using the Kaplan–Meier method. *p*-values < 0.05 were considered significant.

## Results

Out of 239 patients suffering from primary GBM and being treated at our institution during this time period, 73 patients with eloquently located GBM and 35 patients who only underwent biopsy were excluded. One hundred thirty-one patients met the inclusion criteria. Finally, 114 patients were analyzed, because 9 patients did not receive postoperative MRI within 48 h and 8 patients were lost to follow-up. Twenty patients of the 114 patients (18%) were operated on using additional endoscopic assistance. Of these patients, 2/20 (10%) underwent biopsy in an external hospital before but underwent resection in our department before adjuvant treatment. In the entire cohort, 73 patients were male (65%). The mean age was 63.0 ± 13.0 years and 57 patients were older than 65 years (50%).

### Demographic parameters

Age, sex, and location of the tumor were excellently matched, without differences between the two groups. No difference was seen in postoperative KPS (*p*= 0.61). Adjuvant therapies were well balanced. Altogether, nine patients (8.0%) aborted adjuvant treatment (*p*= 0.2). Methylation of the MGMT-status was equally distributed in both groups (*p*= 0.34, Table 1).

**Table 1:** Demographic parameters

	Microscopic approach		Combined approach		Total		*p*-value
	*n*	*%*	*n*	*%*	*n*	*%*	
Sex							
Female	36	38.3	5	25	41	36	0.26
Male	58	61.7	15	75	73	64	
Age							
≤ 65 years	45	47.9	12	60	51	50	0.33
> 65 years	49	52.1	8	40	51	50	
KPS							
≤ 70%	32	34	8	40	40	35.1	0.61
> 70%	62	66	12	60	74	64.9	
GTR							
Yes	63	67.0	20	100	83	72.8	**0.003**
no	31	33.0	0	0	31	27.2	
Location							
Frontal	29	30.9	7	19.4	36	31.6	0.98
Parietal	19	20.2	4	17.4	23	20.2	
Temporal	40	42.6	8	16.7	48	42.1	
Occipital	6	6.4	1	14.3	7	6.1	
Gliadel							
Yes	17	18.1	4	20	21	18.4	0.84
No	77	81.9	16	80	93	81.6	
MGMT status							
Positive	32	34.1	8	40	40	50.0	0.34
Negative	46	48.9	12	60	58	36.0	
Unknown	16	17.0	0	0	16	14.0	
Adjuvante therapy							
Stupp regime	81	86.2	19	95	100	87.7	0.69
Glarius trial	4	4.3	0	0	4	3.5	
CCNU	0	0	0	0	0	0	
RT only	8	8.5	1	5	9	7.9	
None	1	1.1	0	0	1	0.9	
Other	0	0	0	0	0	0	
Therapy aborted	6	6.5	3	15.0	9	8	0.2

Legend: *KPS*, Karnofsky performance scale; *GTR*, gross total resection; *CCNU*, Chlorethyl-Cyclohexyl-Nitroso-Urea (Lomustine); *MGMT*, O 6-methylguanine-*DNA* methyltransferase; *RT*, radiotherapy

Significance level *p* < 0.05

### Extent of resection, progression-free, and overall survival

**Fig. 1:**
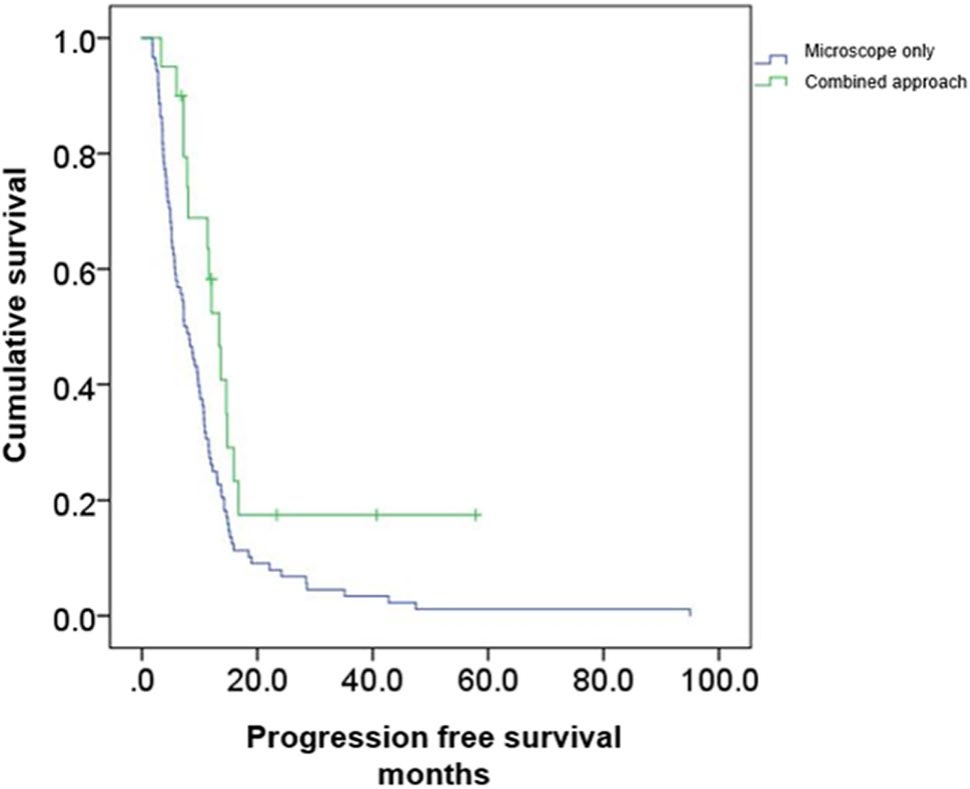
The PFS of the combined approach (green) and the standard microscopic approach (blue) was significantly enhanced when using the assistance of the endoscope [19.3 months (CI95% 10.8–27.7) vs. 10.8 months (CI95% 8.2–13.4; *p*=0.012)]

GTR was achieved in all patients undergoing combined microscope and endoscope-assisted surgery compared to 75.9 % in patients undergoing microscope-assisted surgery only (100% vs. 75.9%, *p*=0.003, Table 1). The PFS in the combined endoscope- and microscope-assisted resection cohort was 19.3 months (CI95% 10.8–27.7) vs. 10.8 months (CI95% 8.2–13.4; *p*=0.012, Fig. 1) in the microscope-assisted resection cohort.

**Fig. 2:**
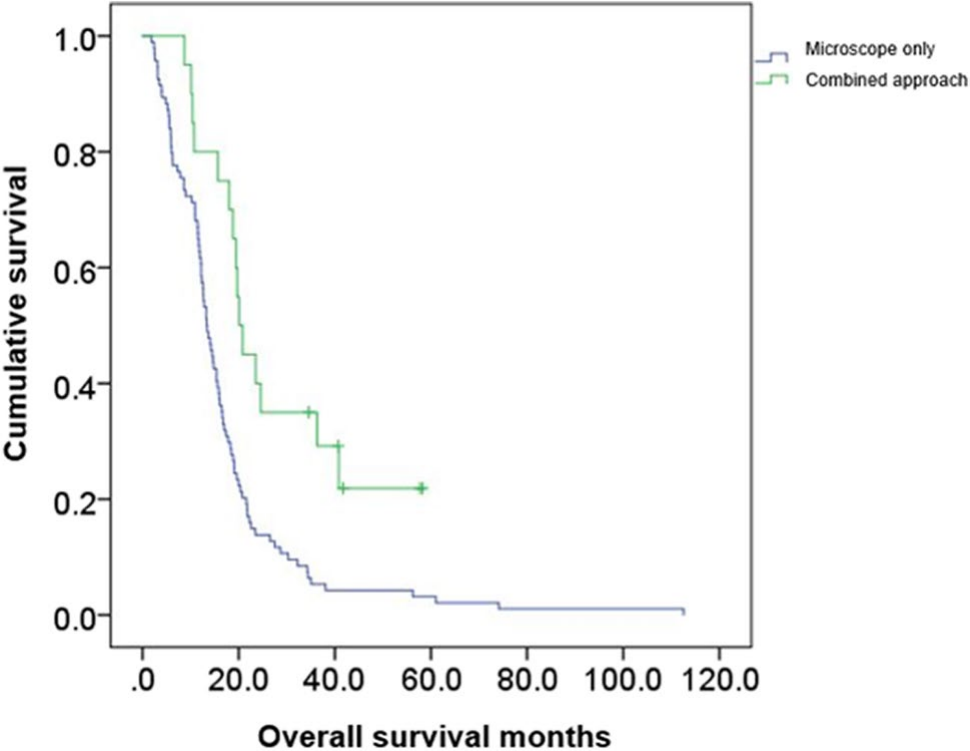
The OS was significantly increased using the combined microscopic/endoscopic approach (green) compared with the standard microsurgical approach [blue, 28.9 months (CI95% 21–37) vs. 16.8 months (CI95% 14–20), *p*=0.001]

OS in the combined endoscope- and microscope-assisted resection cohort was 28.9 months (CI95% 20.4–34.1) compared to 16.8 months (CI95% 14.0–20.9), in the microscope-assisted resection cohort (*p*=0.001, Fig. 2).

## Discussion

In this study, we assessed the effect of combined microscopic and endoscopic fluorescence-guided resection versus standard microscopic fluorescence-guided resection on PFS and OS in patients with non-eloquently located GBM. Our results strongly suggest that PFS and OS can be substantially increased in patients suffering from GBM if endoscopic fluorescence-guided resection was added.

### Effect of combined approach on EOR

Our group recently showed that the assistance of the endoscope enables the surgeon to considerably increase the rate of complete resections (95%) and to achieve a substantially greater EOR, being not limited to the contrast-enhancing parts of the tumor [2, 4]. Furthermore, endoscopic assistance in GBM surgery appears to be safe and feasible as the endoscope identifies tumor tissue with a high sensitivity (100%) and a satisfying specificity (75%) [3]. By significantly reducing the distance between the light source and the tumor tissue, tumor tissue that is microscopically insufficiently visualized (located at the tumor margins, at blind spots around the area of the craniotomy and deeply in the surgical fields with poorer illumination) is detected endoscopically [2, 4, 9, 10]. This in turn leads to increased rates of complete resections and enables supratotal resection instead of GTR. Our results again emphasize the importance of the EOR in GBM surgery and impressively shows that current limitations of standard microscopic fluorescence-guided surgery might be overcome by the assistance of the endoscope.

### Impact on overall survival

The application of 5-ALA has been proven to increase survival in GBM surgery with strong evidence from a prospective randomized neurosurgical trial [12]. In addition, several studies have shown that GTR substantially increases PFS and OS compared with subtotal resection (STR) in patients with GBM [5, 7, 8, 11]. Although our group was able to show, that the endoscope increases the rate of GTR as well as the EOR [2, 4], the impact of the combined approach on survival was still unknown, stimulating the execution of this study. As both cohorts are well balanced regarding possible confounders such as molecular status, adjuvant treatment, performance status, or age, we consider the assistance of the endoscope being capable of increasing the rate of GTR and thereby substantially increasing survival in patients with GBM.

### Strengths and limitations

Our study has several limitations. First, this was a retrospective study and additional prospective studies are required to confirm our results. Second, the cohort being operated using the combined approach is very small and selective. Third, the whole study population is selective as only non-eloquently located GBM were considered. However, both cohorts are well balanced regarding possible confounders, which leads us to suggest that the combined approach is superior to the standard microscopic approach in terms of rate of GTR and survival in patients with GBM.

## Conclusion

This is the first study comparing endoscope-assisted fluorescence-guided GBM resection with a standard neuronavigated microscopic fluorescence-guided resection. The assistance of an endoscope appears to substantially enhance GTR and OS. The strong effect size observed herein is contrasted by the limitations in study design. Therefore, prospective validation is required before we can generalize our findings.
